# Seroprevalence of SARS-CoV-2 antibodies in the poorest region of Brazil: results from a population-based study

**DOI:** 10.1017/S0950268821001163

**Published:** 2021-05-18

**Authors:** Adriano Antunes de Souza Araújo, Lucindo José Quintans-Júnior, Luana Heimfarth, Dulce Marta Schimieguel, Cristiane Bani Corrêa, Tatiana Rodrigues de Moura, Rafael Ciro Marques Cavalcante, Rangel Rodrigues Bomfim, Renata Grespan, Lorranny Santana Rodrigues, Danillo Menezes dos Santos, Ayane de Sá Resende, Nathanielly de Lima Silva, Anna Clara Ramos da Silva Santos, Jéssica Maria Dantas Araújo, Mércia Feitosa de Souza, Marco Aurélio de Oliveira Góes, Victor Santana Santos, Paulo Ricardo Martins-Filho

**Affiliations:** 1 Department of Pharmacy, Federal University of Sergipe, São Cristóvão, Sergipe, Brazil; 2 Health Sciences Graduate Program, Federal University of Sergipe, Aracaju, Sergipe, Brazil; 3 Graduate Program in Pharmaceutical Sciences, Federal University of Sergipe, São Cristóvão, Sergipe, Brazil; 4 Department of Physiology, Federal University of Sergipe, São Cristóvão, Sergipe, Brazil; 5 Department of Morphology, Federal University of Sergipe, São Cristóvão, Sergipe, Brazil; 6 Department of Pharmacy, Federal University of Sergipe, Lagarto, Sergipe, Brazil; 7 Graduate Program in Physiological Sciences, Federal University of Sergipe, São Cristóvão, Sergipe, Brazil; 8 Government of Sergipe State, State Health Secretariat, Aracaju, Sergipe, Brazil; 9 Department of Medicine, Federal University of Sergipe, Lagarto, Sergipe, Brazil; 10 Centre for Epidemiology and Public Health, Federal University of Alagoas, Arapiraca, Brazil; 11 Investigative Pathology Laboratory, Federal University of Sergipe, Aracaju, Sergipe, Brazil

**Keywords:** COVID-19, serioepidemiological studies, severe acute respiratory syndrome coronavirus 2

## Abstract

Population-based seroprevalence studies on coronavirus disease 2019 (COVID-19) in low- and middle-income countries are lacking. We investigated the seroprevalence of severe acute respiratory syndrome-coronavirus-2 (SARS-CoV-2) antibodies in Sergipe state, Northeast Brazil, using rapid IgM−IgG antibody test and fluorescence immunoassay. The seroprevalence was 9.3% (95% CI 8.5–10.1), 10.2% (95% CI 9.2–11.3) for women and 7.9% (IC 95% 6.8–9.1) for men (*P* = 0.004). We found a decline in the prevalence of SARS-CoV-2 antibodies according to age, but the differences were not statistically significant: 0–19 years (9.9%; 95% CI 7.8–12.5), 20–59 years (9.3%; 95% CI 8.4–10.3) and ≥60 years (9.0%; 95% CI 7.5–10.8) (*P* = 0.517). The metropolitan area had a higher seroprevalence (11.7%, 95% CI 10.3–13.2) than outside municipalities (8.0%, 95% CI 7.2–8.9) (*P* < 0.001). These findings highlight the importance of serosurveillance to estimate the real impact of the COVID-19 outbreak and thereby provide data to better understand the spread of the virus, as well as providing information to guide stay-at-home measures and other policies. In addition, these results may be useful as basic data to follow the progress of COVID-19 outbreak as social restriction initiatives start to be relaxed in Brazil.

## Introduction

Coronavirus disease 2019 (COVID-19) caused by severe acute respiratory syndrome-coronavirus-2 (SARS-CoV-2) emerged in late 2019 in China and rapidly spread worldwide leading to a global health outbreak [1]. Until 25 February 2021, COVID-19 has been reported in more than 113 million people worldwide, with the highest number of cases registered in USA, India and Brazil.

Although reverse transcription-polymerase chain reaction (RT-PCR) is considered the gold standard technique for detecting and confirming SARS-CoV-2 infection, RT-PCR has been prioritised for symptomatic people who seek health services, making the exact number of individuals who have been infected by SARS-CoV-2 unknown. Thus, there is large number of asymptomatic cases associated with SARS-CoV-2 that are not reported.

Serological assays provide a more complete picture of infection estimates, including people who having mild or asymptomatic infection or who were never tested despite the symptoms [2]. Moreover, seroepidemiological studies are important to quantify the proportion of the population that remains susceptible to the virus and could be an important indicator for driving decisions by preventing subsequent waves of the COVID-19 outbreak [3].

Although a variety of seroepidemiological studies have been conducted, many of them analysed small and/or non-probabilistic samples leading to limitations in providing precise estimates of seroprevalence by sex and age groups in the general population [4–6]. In Brazil, one of the countries most affected by the COVID-19 outbreak with more than 10 million cases and 250 000 deaths, there is a lack of large-scale diagnostic testing which is critical to controlling the virus in a long term. In this study, we investigated the seroprevalence of SARS-CoV-2 antibodies in 15 municipalities in Sergipe State, Northeast Brazil.

## Methods

### Study design

This was a cross-sectional study consisting of serological testing in individuals not previously tested for SARS-CoV-2 residing in Sergipe state, Northeast Brazil, from 1 July to 31 July 2020.

### Study setting

Sergipe is the smallest state in Brazil and is located in the poorest region of the country. The state is divided into 75 municipalities, has an estimated population of ~2.3 million people, human development index (HDI) of 0.665 and a monthly household income per capita of less than one Brazilian minimum wage (approximately USD 190/month). Until 25 February 2021, SARS-CoV-2 had infected 149 637 people and resulted in 2940 deaths.

### Sampling

Sergipe state is divided into eight administrative health regions. We selected the 15 main municipalities in the state based on the following criteria: the 10 municipalities with the largest population size and the five municipalities that provide access to other states by land. Of the 10 largest municipalities, five are in the metropolitan region (Aracaju, Barras dos Coqueiros, Laranjeiras, Nossa Senhora do Socorro and São Cristóvão) and five outside of the metropolitan area (Capela, Itabaiana, Itabaianinha, Lagarto and Nossa Senhora da Glória). The five municipalities bordering other states were Propriá, Canindé de São Francisco, Simão Dias, Tobias Barreto and Porto da Folha. These 15 municipalities have over 30 000 inhabitants and include approximately 65% of Sergipe population.

We used the following formula to calculate the sample size for each municipality: *n* = *Z*^2^*p*(1–*p*) / *e*^2^, where *n* = sample size; *Z* = 1.96 for a confidence level of 95%; *p* = proportion of individuals exposed to SARS-CoV-2; *e* = margin of error of 5%. For this study, we used *P* = 0.5 to generate the most conservative sample size. A minimal sample size of 384 individuals in each municipality was required. We considered an overall attrition rate of 10% as acceptable and a total of 5615 individuals were included in this study.

In each municipality, households were randomly selected from a sampling frame developed from a census list. At the beginning of the visit, all household members were listed, and one individual was randomly selected for data collection.

### Data collection and procedures

After obtaining written informed consent to participate, individuals were interviewed using a structured questionnaire that included clinical and demographic features. Then, the seroepidemiological survey was conducted in two steps. In the first step, we applied a lateral flow immunoassay (SARS-CoV-2 Antibody Test, Guangzhou Wondfo Biotech Co., Ltd.) using fingerstick blood specimen to detect IgM/IgG antibodies against SARS-CoV-2 within 15 min. This rapid IgM/IgG combined antibody test has been widely used in screening asymptomatic SARS-CoV-2 carriers [7] and a weakly positive band can be considered as a positive result according to the manufacturers' instructions. The interpretation of this test was carried out by two trained researchers to minimise bias on the visualisation of the line for IgM/IgG and only consensus results were recorded. The sensitivity and specificity for this point-of-care test are 86.4% and 99.6%, respectively, according to the manufacturer when assessed on 361 confirmed SARS-CoV-2 patients and 235 negative controls (https://testecovid19.org/faq/one-step-covid-2019-test-guangzhou/).

**Fig. 1.**
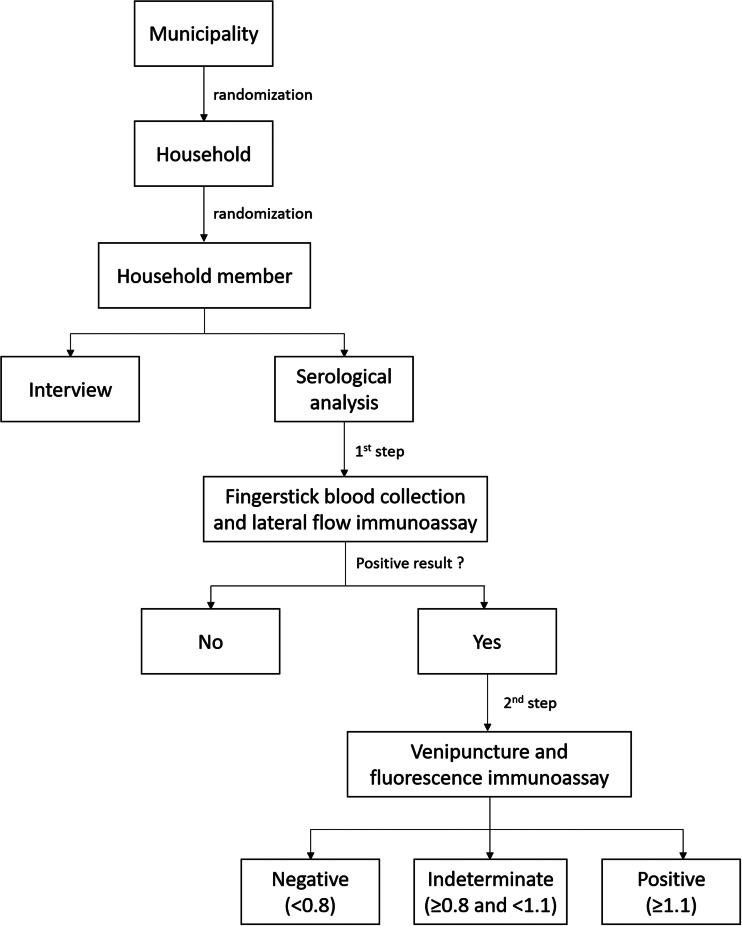
Flowchart showing the major steps involved in conducting the study.

In the second step, venous blood from individuals tested positive in the screening step was collected aseptically using venipuncture and a fluorescence immunoassay (FIA) (iChroma II, BioSys + Kovalent) for qualitative detection and differentiation of IgM and IgG antibodies against SARS-CoV-2 was performed. A result was considered negative if the automated reader obtained a readout <0.8, indeterminate if ≥0.8 and <1.1 and positive if ≥1.1. The sensitivity and specificity of the FIA are 95.8% and 97.0%, respectively, according to the manufacturer when assessed on 46 SARS-CoV-2 positive patients and 131 negative controls (http://www.biosys.com.br/wp-content/themes/transport/covid-19-images/SOLUCAOPOCT-COMPLETA-COVID-19.pdf). This test output is a numerical value and do not exhibit personal interpretation bias. A flowchart showing all steps and procedures is shown in Figure 1.

### Outcome

The main outcome was seroprevalence expressed as the proportion of individuals who had a positive result in the FIA.

### Data analysis

Seroprevalence with 95% confidence interval (CI) was calculated by using the Wilson procedure [8] and estimated according to age (0–19, 20–59 and ≥60 years), sex and geographic region (within metropolitan area and other municipalities). Pearson's chi-square tests were performed to examine differences in seroprevalence by sex and geographic location. The Mantel−Haenszel test for trend was used to evaluate changes in seroprevalence according to age. All tests were two-sided at the 5% significance level. Analyses were performed by using R software (version 3.5.3; R Foundation for Statistical Computing, Vienna, Austria).

### Ethical considerations

This study was approved by the Ethics Committee of the Federal University of Sergipe (protocol number 33095120.4.0000.5546). Written informed consent was obtained from all study participants and positive cases were reported to the Municipal Health Secretariats.

## Results

Of the 5615 individuals enrolled, 3353 (59.7%) were women and 2262 (40.3%) were men. The median age was 42 years (interquartile range (IQR): 28–57 years). Age data were missing for 40 (0.7%) participants. A total of 1909 (34.0%) individuals lived within the metropolitan area and 3706 (66.0%) outside the metropolitan area.

In the screening step, 652 had a positive rapid IgM/IgG combined antibody test. In the second step, venipuncture was performed in 619 out of 652 individuals and 520 had a positive result in the FIA. Five hundred out of 520 (96.2%) positive samples had detectable levels of SARS-CoV-2 IgG, 18 (3.4%) both IgM and IgG SARS-CoV-2 antibodies and two samples (0.4%) had detectable levels of SARS-CoV-2 only for IgM. Of the 520 individuals with SARS-CoV-2 antibodies detected using the FIA-based method, 342 (65.8%) were women, the median age was 41 years (IQR: 28–56 years) and 223 (42.9%) lived within the metropolitan area.

**Fig. 2.**
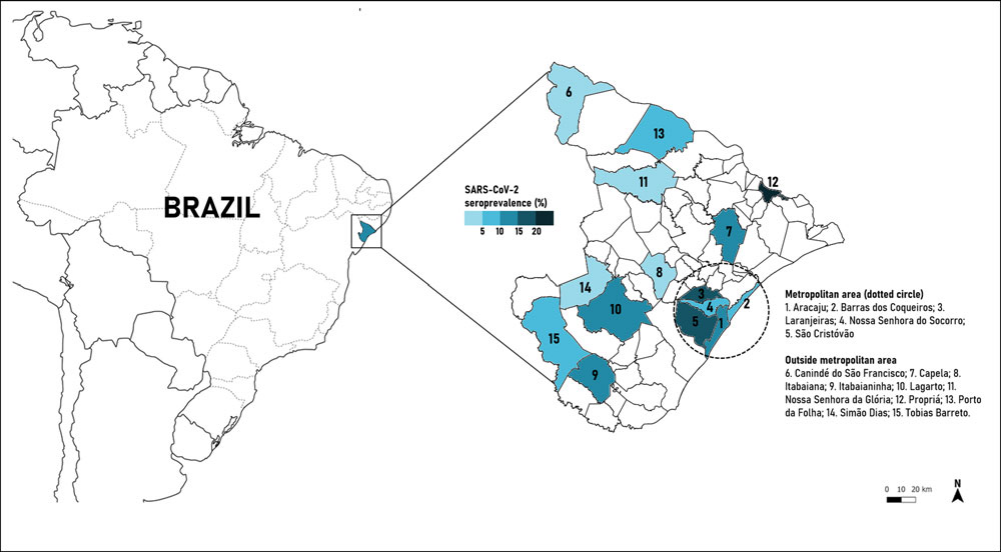
Mapping the SARS-CoV-2 seroprevalence in Sergipe state, Northeast Brazil.

The seroprevalence of SARS-CoV-2 antibodies was 9.3% (95% CI 8.5–10.1), 10.2% (95% CI 9.2–11.3) for women and 7.9% (IC 95% 6.8–9.1) for men (*P* = 0.004). We found a decline in the prevalence of SARS-CoV-2 antibodies according to age, but the differences were not statistically significant: 0–19 years (9.9%; 95% CI 7.8–12.5), 20–59 years (9.3%; 95% CI 8.4–10.3) and ≥60 years (9.0%; 95% CI 7.5–10.8) (*P* = 0.517). The metropolitan area had a higher seroprevalence of SARS-CoV-2 (11.7%, 95% CI 10.3–13.2) than other municipalities (8.0%, 95% CI 7.2–8.9) (*P* < 0.001) (Table 1 and Fig. 2).

**Table 1.** Results of point-of-care lateral flow and fluorescence immunoassays for SARS-CoV-2 antibodies

Municipalities	Sample	Positive results according to the rapid test (%)	Fluorescence immunoassay	
			Positive results	Seroprevalence of SARS-CoV-2 antibodies (%) (95% CI)
Metropolitan area	1909	261 (13.7%)	223	11.7 (10.3–13.2)
Aracaju	422	49 (11.6%)	42	10.0 (7.5–13.2)
Barra dos Coqueiros	382	40 (10.5%)	34	8.9 (6.4–12.2)
Laranjeiras	358	64 (17.9%)	57	15.9 (12.5–20.1)
Nossa Senhora do Socorro	378	39 (10.3%)	29	7.7 (5.4–10.8)
São Cristóvão	369	69 (18.7%)	61	16.5 (13.1–20.7)
Outside metropolitan area	3706	391 (10.6%)	297	8.0 (7.2–8.9)
Canindé de São Francisco	351	11 (3.1%)	6	1.7 (0.8–3.7)
Capela	358	51 (14.2%)	38	10.6 (7.8–14.2)
Itabaiana	376	14 (3.7%)	10	2.7 (1.5–4.8)
Itabaianinha	410	55 (13.4%)	43	10.5 (7.9–13.8)
Lagarto	387	74 (19.1%)	54	14.0 (10.9–17.8)
Nossa Senhora da Glória	361	29 (8.0%)	16	4.4 (2.8–7.1)
Propriá	342	78 (22.8%)	69	20.2 (16.3–24.8)
Porto da Folha	389	44 (11.3%)	32	8.2 (5.9–11.4)
Simão Dias	354	5 (1.4%)	5	1.4 (0.6–3.3)
Tobias Barreto	378	30 (7.9%)	24	6.4 (4.3–9.3)
Total	5615	652 (11.6%)	520	9.3 (8.5–10.1)

## Discussion

This household-based survey investigated the seroprevalence of SARS-CoV-2 antibodies in a population 16−20 weeks after confirmation of the first case of community transmission of SARS-CoV-2 infection in Sergipe, Northeast Brazil. Overall, the SARS-CoV-2 seroprevalence in Sergipe was 9.3%, which is higher than the prevalence reported in several geographic regions worldwide [9]. Our findings also showed a higher prevalence of SARS-CoV-2 antibodies among women and within the metropolitan area.

A nationwide study performed in the USA between 23 March and 12 May 2020, using enzyme linked immunosorbent assay (ELISA) showed that seroprevalence of SARS-CoV-2 ranged from 1.0% (95% CI 0.3–2.4) in San Francisco Bay area to 6.9% (95% CI 5.0–8.9) in New York [2]. In Spain, a serological survey using chemiluminescent immunoassay carried out between 27 April and 10 May 2020, showed a prevalence of SARS-CoV-2 antibodies for the entire country of 4.6% (95% CI 4.3–50), but in seven provinces in the central part of Spain, including Madrid, the seroprevalence was greater than 10% [3]. Similar results were found in a Swiss study which found a seroprevalence about 11% in the general population of Geneva during the first week of May 2020 [10]. Contrasting findings were observed in serosurveys performed in Wuchang district, Wuhan, China [11], 70 districts of the 21 states of India [12] and Southern Brazil [4] where the prevalence of SARS-CoV-2 antibodies was less than 2.5% in May 2020.

Recently, a nationwide study using a lateral flow point-of-care test found a SARS-CoV-2 antibody prevalence in the Northeast Brazil of 3.2% (95% CI 2.8–3.7%) during the first week of June. Seroprevalence estimates found in Aracaju (Sergipe state capital) and Itabaiana (the largest municipality outside the metropolitan area) were 0.9% (95% CI 0.1–3.2%) and 1.4% (95% CI 0.3–3.9%), respectively [13]. Interestingly, although the seroprevalence in Itabaiana was similar to the estimated in the present study (2.7%), there is a significant divergence in the results of the Aracaju municipality (10.0%), which may be related especially to the sudden increase in cases of COVID-19 in the city during July 2020, considering the peak of the outbreak in the state during the first wave. In addition, it has been found that wide variations in positivity rate of SARS-CoV-2 infection can be influenced by the target population, sample size, socio-economic variables, cultural practices, political decision-making, interventions to mitigate spread of SARS-CoV-2, health infrastructure and diagnostic performance of serology assay [9].

The seroprevalence of SARS-CoV-2 found in Sergipe state was distributed heterogeneously according to geographical location, with higher prevalence in metropolitan area than other municipalities. This supports the findings of previous studies which showed higher rates of SARS-CoV-2 infection in populations living in highly dense areas compared to areas with a smaller population [3, 10, 14, 15]. Metropolitan areas often have higher traffic of people increasing the likelihood of a healthy person enter in contact with an infected person (symptomatic or asymptomatic) and thus facilitating the spread of the virus. In addition, there is emerging evidence showing that people living in socio-economically disadvantaged neighbourhoods in Aracaju have been disproportionately impacted by COVID-19 which contributed to the increase in the number of cases and deaths in this region [16].

A recent systematic review and meta-analysis suggested no differences on the seroprevalence of SARS-CoV-2 according to sex and age [9]. However, the results of the present study are contrasting. We found a decline in seroprevalence with increasing age despite differences were not statistically significant. The lower seroprevalence estimates among older adults in Sergipe could be associated with the social distancing measures and are a sign that targeted efforts to reduce social mixing of these people with others might have succeeded. The declining antibody detection rate by age can be a useful tool for tracking vaccine priority for population since the herd immunity is a challenge that the vaccination programmes must overcome to reduce cases, hospitalisations and deaths related to COVID-19 and the burden on health systems [17]. Furthermore, our findings showed a higher prevalence of SARS-CoV-2 infection among women similar to that described in Wuhan (China) [11], Louisiana, Minnesota and Utah (USA) [2]. Gender differences in the SARS-CoV-2 prevalence have been debated worldwide and may be influenced by cultural [18] and unmeasured confounding factors including social and workplace exposures, interactions and behaviours [19].

This study has some limitations and included especially the high probability of false-negative results though point-of-care tests are mainly within the first two weeks after SARS-CoV-2 exposure. The available evidence about the diagnostic accuracy for COVID-19 serological tests has been characterised by high risks of bias, heterogeneity and limited generalisability to point-of-care testing and to outpatient populations [20]. However, in a recent study it was found that Wondfo^®^ SARS-CoV-2 Antibody Test had the highest sensitivity and specificity results among the lateral flow immunoassays [21]. Moreover, although the findings of rapid tests may be affected by operator mistake in interpreting the results, this bias was minimised by a double check analysis performed by two trained people.

In summary, this study showed that the SARS-CoV-2 seroprevalence in Sergipe state, Northeast Brazil, after 16−20 weeks of the first case of COVID-19 was estimated to be 9.3% with higher rates in metropolitan area, among women, and a trend to decrease with age. These findings highlight the importance of serosurveillance to estimate the real impact of the COVID-19 outbreak and thereby offer data to better understand the spread of the virus, as well as providing information to guide stay-at-home measures and other policies. In addition, these results may be useful as basic data to follow the progress of COVID-19 outbreak as social restriction initiatives start to be relaxed in Brazil.

## Data Availability

The data that support the findings of this study are available from the corresponding author, upon reasonable request.
